# Gait pattern in patients treated with a total hip arthroplasty due to an acute displaced cervical neck fracture: a randomised comparison between 29 cases with a cemented femoral stem and 16 cases with an uncemented femoral stem

**DOI:** 10.1177/11207000231208099

**Published:** 2023-12-12

**Authors:** Roland Zügner, Roy Tranberg, Bita Sharegi, Johan Kärrholm

**Affiliations:** Department of Orthopaedics, Institute of Clinical Sciences, Sahlgrenska Academy, University of Gothenburg, Sahlgrenska University Hospital, Göteborg, Sweden

**Keywords:** Cervical femur fracture, gait, hip, hip arthroplasty, imaging, randomised, three-dimensional

## Abstract

**Background:**

The choice between cemented or uncemented stem fixation in the treatment of a femoral neck fracture may influence patient rehabilitation and the resulting gait pattern, due to potential differences in implant positioning and fixation. We used gait analysis to study temporal gait parameters, hip kinematics and kinetics in patients who, 2 years previously, had been randomised to treatment with a cemented or uncemented stem and due to an acute femoral neck fracture.

**Methods:**

45 Patients implanted with a cemented Lubinus SP II (*n* = 29) and an uncemented (*n* = 16) Corail stem were studied. Gait analysis was performed using a 16-camera motion capture system and force plates. 28 subjects served as controls. Temporal gait parameters, hip kinematics and kinetics were analysed. The patients had no or minimum pain (median Harris pain score 44, range 40–44) and the majority had no limp (median Harris limp score 11, range 5–11).

**Results:**

Temporospatial gait parameters and abduction-adduction motions and moments did not differ between patients with cemented or uncemented stems (*p* *>* 0.05). Patients with cemented stems did, however, show more hip flexion and less extension during walking than those with an uncemented stem (*p* < 0.05). Moreover, the flexion-extension range was less in the cemented group (*p* < 0.04). Compared with controls, the hip fracture patients walked more slowly, with a shorter stride length and a longer stance phase.

**Conclusions:**

Increased hip flexion and reduced extension in patients using the Lubinus SP II cemented stem could be an effect of its anteverted neck, but this question requires further study. Despite acute treatment with THA, hip fracture patients demonstrated a change in gait pattern compared with controls 2 years after the operation. This suggests that these changes are caused by the presence of an implant, or the soft-tissue trauma partly caused by the surgery than by any degenerative disease present in patients undergoing elective surgery.

ClinicalTrials.gov Identifier: NCT04791605

## Introduction

A displaced femoral neck fracture is a common injury in the elderly population, and it is treated with a hemi- or a total hip arthroplasty (THA) as standard practice. Surgery with THA is often chosen for somewhat younger, more independent patients with longer life expectancy. The use of cemented fixation has been associated with better functional results up to 1 year after surgery,^1,2^ a reduced risk of periprosthetic fracture,^3–7^ and no certain increased mortality when compared with uncemented fixation.^2,8^

The objective recording of the walking pattern using an optical tracking system (OTS) is a useful complement to other evaluation instruments such as patient-reported outcome measurements (PROMs).^9^ Previous studies of joint motions during gait in patients with THA have consistently reported less hip extension, hip flexion/extension range, reduced speed and stride length, in addition to peak hip abduction moment during stance.^10–13^ Further, hip joint motions during gait in patients with THA have shown greater variability compared with controls.^13^ These studies were performed in patients undergoing elective THA due to various hip diseases, mainly osteoarthritis, which means that the range of motion and muscular function were influenced in various ways before the THA was inserted.

Our main purpose was to evaluate the gait performance 2 years after an acute displaced femoral neck fracture in patients randomised to either cemented or uncemented fixation of the stem. Temporospatial gait parameters, kinematics and kinetics were recorded. We hypothesised that the use of stem fixation had no influence on the observed gait performance. As a secondary purpose, we evaluated whether patients without a previous hip disease who due to hip fracture, received an acute THA would display less pronounced deviations from normal than patients with an elective THA based on a comparison with previously published data.

## Methods

**Figure 1.**
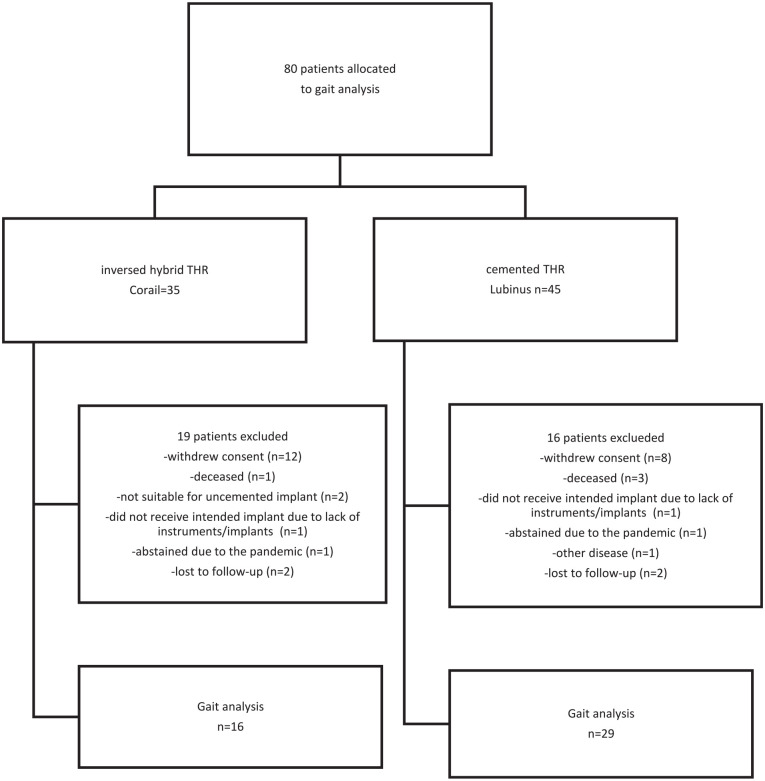
A CONSORT diagram showing the patients included in the study.

Between 2010 and 2018 100 patients with a displaced femoral neck fracture (Garden III–IV) referred to Sahlgrenska University Hospital, Mölndal, Sweden, were recruited to a study of influence of stem fixation on the outcome with respect to postoperative confusion as primary outcome. The patients were randomly allocated with use of sealed envelopes, to receive either a cemented Lubinus SP II stem and a cemented IP acetabular cup (Link, Germany) (*n* = 45) or an uncemented Corail stem and a cemented Marathon cup (Depuy, USA) (*n* = 35). To become included the patient should come from their own home and should have been independent walkers before fracture. Patients with known neurological disease, dementia or with any gait disability before fracture were excluded. 80 of the patients (45 randomised to a cemented and 35 with an uncemented stem) were asked to participate in the evaluation of the gait pattern 2 years after the operation. The first 20 patients were not asked to participate because they had already past 2 years when the ethical approval was available. Among the 80 patients included, one in the Corail group had received an uncemented Trilogy (Zimmer) instead of a Marathon cup due to the unavailability of devices used for cemented cup fixation at the time of surgery. All the patients received a 32-mm cobalt-chromium femoral head (Figure 1).

16 patients in the cemented and 19 patients in the uncemented stem group did not want to or could not attend. For 2 patients in each group the reason for not to attend was unknown. They were assigned lost to follow-up (Figure 1). Thus, 29 patients in the Lubinus SP II group and 16 patients in the Corail group successfully completed 1 gait analysis session. This patient cohort consisted of 28 females and 17 males with a median age of 74 years (range 64–87 years). Patient demographics, at baseline, are presented in Table 1. The patients (45 hips) in the current study were operated on by 27 surgeons, corresponding to one to four interventions per surgeon. All the surgeries were performed within 36 hours after the fracture using a lateral approach.

**Table 1.** Descriptive data of included patients and controls Harris Hip Score (HHS) for pain and limping.

	Corail *n =* 16		Lubinus SP II *n =* 29		Controls *n =* 28		Corail vs. Lubinus SP II	Comparison with controls	
								Corail	Lubinus SP II
	Median	Range	Median	Range	Median	Range	*p*-value^a^	*p*-value^a^	*p*-value^a^
Age	74	64–87	74	66–87	60	55–84	0.64	***<0.001***	***<0.001***
Height	1.74	1,56–1.82	1.69	1.52–1.96	1.73	1.54–1.84	0.35	0.32	0.89
Weight	75.2	62–108	74	39–115	75	50–97	0,20	0.43	0.58
BMI	27.2	21.3–34.8	25.2	16–34.5	26.3	21–31	0.19	0.38	0.45
HHS pain	44 *n =* 13	40–44	44 *n =* 28	40–44			1.0		
HHS limp	11 *n =* 13	8–11	11 *n =* 28	5–11			0.7		

BMI, body mass index; HHS, Harris Hip Score.

Values in bold indicate statistical significance.

a Mann-Whitney U-test.

Clinical outcome was evaluated using the Harris Hip Score (HHS) and the sub-scores for pain and limping for 28 patients in the Lubinus group and for 13 patients in the Corail group at the 2-year follow-up.

28 persons recruited from staff, relatives and students were included as a healthy control for comparison (Table 1). The control subjects were included if they had no history of orthopaedic or neuromuscular diseases that could influence their walking ability. There were no differences between the study groups and the healthy group regarding height, weight and body mass index (BMI). However, the study group consisted of participants with a higher median age than in the control group, *p* = 0.001.

The development of radiolucent lines and stem subsidence was studied on conventional radiographs (anteroposterior [AP] and lateral). The postoperative examination and the one performed at 2 years were used. The extent of any radiolucent line between the cement and bone or between the stem and bone was measured using dedicated software (MDesk, RSA Biomedical, Umeå, Sweden). The length of radiolucent lines relative to the total length of the interface was computed by software. We also measured the proximal-distal translation of the femoral head centre using the lesser trochanter as a bony landmark. In the individual case, a translation exceeding 5 mm was regarded as representing a true value.^14^ All the measurements were performed by 1 of the authors (JK).

The gait analysis was performed using an optical tracking system (OTS). Dressed in shorts and a sleeveless shirt, the patients walked barefoot on the floor at a self-selected speed. Prior to the recordings, 15 spherical markers (ø 12 mm) were attached to the skin with double-sided adhesive tape on the lower extremities and the pelvis by an experienced examiner (RZ). The skin marker model has previously been presented and has been tested for reliability and validity.^9,13,15,16^ Markers were attached to prominent landmarks between the second and third metatarsals at the lateral malleolus, tuber calcanei, tibial tubercle, proximal border of the patella, lateral knee joint line, border of the sacrum and the anterior and superior iliac spine. A modified Coda pelvis was used to define the pelvis segment. This modification included a reduction of the 2 bilateral markers on the posterior superior iliac spine to only 1 marker placed at the mid-point between the most proximal border of the sacrum.^17^

A 16-camera motion capture system with a sampling rate of 240 Hz (Oqus 700+, Qualisys AB, Göteborg, Sweden), together with 4 force plates (Amti OPTIMA-High Performance Series (HPS400600) AMTI Watertown, MA 02472, USA), was used for data acquisition. A static recording with the test patient standing in an upright position in the calibrated volume aligned to the global co-ordinate system was performed prior to the gait analysis to scale the subject’s anthropological measurements in relation to the marker positions. The patient was then asked to walk 5–10 times at a self-selected speed through the calibrated volume to familiarise themselves with the situation and then to perform 6 gait trials of which the approved trials for each test subject were selected for further evaluation. The mean values of all the approved trials for each patient were used in the analysis to increase the reliability of the testing. A trial was excluded from the analysis if the patient did not place the step within the border of the force plates correctly or due to other technical problems. The spatiotemporal variables that were collected were speed (m/s), step length (m) and the stance phase percentage of total gait cycle. The kinematic variables were expressed as hip extension and flexion, adduction, and abduction. Kinetic variables collected in the frontal plane during the stance phase were moment (Nm/kg) and power (W/kg) in the hip joint. Prior to any calculations, the marker data obtained from the recordings were filtered using a Butterworth 4th filter with a cut-off frequency of 6 Hz. For calculations of spatiotemporal gait variables, kinematic and kinetic variables, Visual 3D software (C-Motion, Inc., Germatown, USA) was used.

All the statistical analyses were performed using IBM SPSS version 23.0.0 software (IBM SPSS, New York, USA). Descriptive statistics were used to describe the patient characteristics and outcomes. A Shapiro-Wilk test for normality was performed to determine whether the data were normally distributed. Since not all the variables were normally distributed, the Mann-Whitney U-test was used. The level of significance was set at *p* ⩽ 0.05.

The current study was approved by the ethical review board in Gothenburg (reference number 559-09, 2010-02-23). All the patients received oral and written information about the study and were informed of their right to withdraw from the study at any time without explanation. All the patients signed a written consent to participate according to the World Medical Association Declaration of Helsinki and ICMJE Recommendations for the Protection of Research Participants.

## Results

At 2 years, the HHS, pain and limp score, did not differ between the study groups (Table 1) (*p* ⩾ 0.7), missing data in 1 patient with a Corail stem and 3 with a Lubinus SP II stem). The median pain score was 44 (range 40–44) for both groups. The corresponding median limp score was 11 for both groups (Corail: range 8–11; Lubinus: 5–11). Missing values for the Lubinus group were (*n* = 1) and the Corail group (*n* = 3).

At 2 years, the median proximal (+) – distal (−) translation of the femoral head centre was 0 mm (range 5–(−4) mm) in the Corail and 0 mm (5–(−3) mm) in the SP II group (*p* = 0.7). The median relative length of radiolucent lines on the AP and lateral views was zero in both groups (range Corail/Lubinus SP II, AP view: 0–21/0–28%; lateral view: 0–22/0–30%, *p* *>* 0.22). In both groups, the median proximal-distal translation of the femoral head centre was zero (range −4–5/−3–5, *p* = 0.7). In 1 case (Corail), measurements of femoral head centre translations could not be made due to the poor quality of the postoperative radiographs.

Patients with a cemented Lubinus SP II stem showed 4–5° more hip flexion and about 7 degrees less extension than the patients with an uncemented Corail stem (*p* < 0.05). The mean range of hip flexion-extension was almost 7° more in the uncemented group (*p* < 0.05). The adduction/abduction hip kinematics (angles) or kinetics (moments and power) did not differ (*p* ⩾ 0.10) (Table 1).

Compared with controls, both patient groups walked with a lower speed, shorter stride length and longer stance phase (*p* ⩾ 0.005). The hip flexion/extension range was smaller in both patient groups, as was hip flexion in the group with Corail stems (*p* ⩾ 0.009) and hip extension in both groups (*p* ⩽ 0.02). Moreover, in the frontal plane, the hip adduction was lower, with significant values for the Lubinus SP II group (*p* = 0.01). The range of hip abduction/adduction was also smaller in both patient groups (*p* = 0.001) due to reduced abduction (*p* < 0.001). Both groups displayed higher mean adduction, but this was only significant for the group with a cemented Lubinus SP II stem (*p* = 0.01).

The hip adduction/abduction moment range in the frontal plane was significantly higher in both patient groups compared with controls, with a significantly lower adduction moment in the Lubinus group (*p* = 0.001). Generated abduction power and absorbed adduction power were significantly higher in the patient groups (Table 2).

**Table 2.** Gait parameters in the patient groups (Corail and Lubinus SP II stem) at the 2-year follow-up. together with controls.

	Corail *n =* 16		Lubinus SP II *n =* 29		Controls *n =* 28		Corail vs Lubinus SP II	Comparison with controls	
								Corail	Lubinus SP II
	Mean	95% C.I.	Mean	95% CI	Mean	95% CI	*p*-value^a^	*p*-value^a^	*p*-value^a^
Speed (m/s)	*0.89*	*0.71–1.07*	*0.92*	*0.82–1.03*	*1.18*	*1.11–1.24*	*0.71*	***0.003***	***<0.001***
Step length (m)	*1.06*	*0.93–1.20*	*1.00*	*0.90–1.10*	*1.30*	*1.26–1.35*	*0.46*	***<0.001***	***<0.001***
Cadence (steps/min)	*100.7*	*90.5–110.8*	*107.4*	*102.9–111.8*	*100.2*	*93.4–107.0*	*0.19*	*0.98*	*0.055*
Stance (%)	*66.3*	*63.3–69.4*	*63.0*	*61.5–64.4*	*61.3*	*60.5–62.1*	*0.06*	***0.002***	***0.053***
Hip extension ()*degree*	*-6.4*	*-12.1–(-0.7)*	*0.7*	*-3.1–4.5*	*-12.2*	*-14.2–(-10.2)*	***0.049***	***0.02***	***<0.001***
Hip flexion *degree*	*23.7*	*20.2–27.2*	*29.4*	*26.1–32.8*	*29.1*	*27.2–31.0*	***0.023***	***0.009***	*0.87*
Hip ext-flex range *degree*	*29.6*	*12.6–42.2*	*22.8*	*14.1–36.9*	*41.3*	*39.3–43.4*	***0.041***	***<0.001***	***<0.001***
Hip adduction *degree*	*5.1*	*3.1–7.1*	*5.8*	*4.4–7.2*	*3.2*	*2.1–4.4*	*0.55*	*0.11*	***0.01***
Hip abduction *degree*	*-1.0*	*-2.7–0.6*	*-0.8*	*-2.2–0.6*	*-6.2*	*-7.6–(-4.8)*	*0.66*	***<0.001***	***<0.001***
Hip add-abd range *degree*	*7.3*	*2.1-9.5*	*10.7*	*2.2–13.0*	*9.4*	*8.1–10.7*	*0.85*	***0.001***	***0.001***
Hip adduction moment *Nm/kg*	*-0.18*	*-0.26–(-0.11)*	*-0.10*	*-0.14–(-0.06)*	*-0.35*	*-0.46–(-0.24)*	*0.10*	*0.11*	***<0.001***
Hip abduction moment *Nm/kg*	*0.74*	*0.64–0.83*	*0.72*	*0.63–0.81*	*0.71*	*0.59–0.84*	*0.90*	***<0.001***	*0.84*
Hip add-abd moment range *Nm/kg*	*1.1*	*0.0–1.1*	*1.1*	*0.1–1.2*	*1.1*	*1.0–1.1*	*0.86*	***0.001***	***0.001***
Hip abd-absorbed power	*-0.31*	*-0.41–(-0.21*	*-0.36*	*-0.51–(-0.22)*	*-0.57*	*-0.65–(-0.48)*	*0.85*	***<0.001***	***<0.001***
Hip abd-generated power	*0.25*	*0.16–0.34*	*0.29*	*0.20–0.37*	*0.06*	*0.05–0.08*	*0.66*	***<0.001***	***<0.001***

CI, confidence interval.

Values in bold indicate statistical significance.

a Mann-Whitney U-test.

## Discussion

Previous studies of hip fracture patients with use of gait analysis are few and have mainly focused on treatment with various types of osteosynthesis or compared osteosynthesis with hip arthroplasty treatment.^18,19^ Pfeufer et al.^20^ compared patients treated with THA due to a femoral neck fracture with a group of patients treated with osteosynthesis due to per trochanteric fracture. They found that the patients treated with hip arthroplasty generated higher peak force during loading but did not report any kinematic parameters.

We found reduced speed, step length and stance during gait in patients undergoing surgery with THA because of a displaced femoral neck fracture regardless of using cemented or uncemented fixation and compared with a control population. Less hip extension and a lower flexion/extension range and abduction and adduction/abduction range were observed, as well as significant changes in the adduction/abduction moment range and absorbed and generated power in the frontal plane during stance.

The 2 treatment groups did not differ regarding spatiotemporal gait parameters, but the group with cemented fixation showed less hip extension and more hip flexion than that observed in the uncemented group. The reason for this difference in the hip joint motions in the sagittal plane is unknown. It is possible that the anteverted design of the Lubinus SP II stem moves the arc of motion into more flexion and less extension, but this theory requires further confirmation.

Both groups showed a slightly increased hip adduction angle in the frontal plane, but this was only significant for the Lubinus SP II group and both groups had reduced abduction. The reason for this tendency to place the operated hip in a more flexed and adducted position is not known, but it could be a way to compensate for impaired balance and/or loss of strength in the abductor musculature after surgery. Since all the hip joints were operated on using a direct lateral approach, we do not know the extent to which this deviation from normal relates to the presence of an artificial joint or the extent of the soft-tissue trauma caused by the operation itself. As opposed to the situation in elective surgery where the joint and soft tissues have deteriorated for a period often extending to years, the soft-tissue envelope in our cases with a fracture has only been subjected to degenerative changes caused by age, which suggests that the observed changes are mainly caused by the operation itself. Nor did the clinical results as judged by the Harris Hip Score differ between the study groups, which also suggests that the observed differences between the study groups and the controls are related to differences in age and/or the surgical procedure.

There are several limitations to our study due to many dropouts, mainly because we wanted to avoid the bias caused by other degenerative diseases or previous joint arthroplasty in the lower extremities. Some patients did not wish to participate, suggesting selection bias, albeit outside our control. Many surgeons were involved, but all of them had long experience of THA or were assisted by a colleague with this experience. The controls were 14 years younger on average than the participants in the 2 study groups, which might have influenced the results. Oberg et al.^21,22^ observed reduced walking speed and step length with increasing age, which could explain our findings to a certain extent. Age-related changes in joint angles were, however, found to be small and inconsistent. The age-related data for joint angles were therefore regarded as unnecessary in comparative studies.^21,22^

In contrast to patients with osteoarthritis, the patients in our study had according to our knowledge not had any hip disease before fracture, with more or less pronounced influence on mobility and gait. As a result, no preoperative adaptation of the walking pattern due to pain, contracture, and posture could be expected as in patients with osteoarthritis of the hip. Further, the radiographic evaluation indicated that all the stems were stable. We therefore think it reasonable to expect that the gait pattern in our study group of fracture patients had reached a steady state at the time of the gait analysis in agreement with previous observations of patients with primary osteoarthritis.^13,23^ It might be that an artificial joint impairs the feedback from the sensory-neurological system and the opportunity to co-ordinate or fine-tune hip movements while walking.^23^

Our group has previously observed that walking speed and hip extension improved in cases with THA when compared with patients with unilateral hip osteoarthritis. Further, hip flexion/extension and the hip abduction/adduction range turned out to be somewhat better in the operated group but still not normal.^13^ The reason why patients who have been treated with elective THA and despite good or excellent clinical results do not resume a normal walking pattern is not known. A long period of walking disability enabling the development of contracture and changed muscular activation is, however, a reasonable assumption.

All the fracture patients in our study came from independent living. Fracture patients generally constitute a more fragile part of the population, which might influence their gait to a certain extent. We assume, however, that these circumstances and especially the fact that we compared them with osteoarthritic patients only marginally influenced their gait before the operation.

## Conclusion

We found significant differences between patients with a femoral neck fracture treated with a cemented or an uncemented stem. These differences in terms of increased hip flexion and reduced extension with use of a cemented stem could be an effect of choice of fixation. We think, however, that they in whole or in part could be explained by the use of an anterverted stem in the cemented group, a hypothesis that needs to be studied more specifically.

As a spinoff of this study, we also found that despite the presence of a normal hip before fracture, our fracture patients showed fairly pronounced deviations in gait compared with controls, 2 years after the operation. Similar deviations have previously been observed in patients operated with hip arthroplasty due to osteoarthritis. This suggests that the observed deviations from normal gait to a certain extent could be caused by surgery and/or the presence of an implant. In cases of femoral neck fracture, the soft-tissue trauma at the time of the accident could also contribute.
